# The Effects of the Levosimendan Metabolites OR-1855 and OR-1896 on Endothelial Pro-Inflammatory Responses

**DOI:** 10.3390/biomedicines11030918

**Published:** 2023-03-16

**Authors:** Hannah Kipka, Rebecca Schaflinger, Roland Tomasi, Kristin Pogoda, Hanna Mannell

**Affiliations:** 1 Doctoral Program Clinical Pharmacy, University Hospital, LMU Munich, 81377 Munich, Germany; 2 Institute of Cardiovascular Physiology and Pathophysiology, Biomedical Center, LMU Munich, 82152 Planegg, Germany; 3 Department of Anaesthesiology, University Hospital, LMU Munich, 81377 Munich, Germany; 4 Physiology, Institute for Theoretical Medicine, University of Augsburg, 86159 Augsburg, Germany

**Keywords:** levosimendan, OR-1896, OR-1855, endothelial cells, reactive oxygen species, inflammation

## Abstract

The calcium sensitizer levosimendan is used for the treatment of acute decompensated heart failure. A small portion (4–7%) of levosimendan is metabolized to the pharmacologically active metabolite OR-1896 via the inactive intermediate OR-1855. In addition, levosimendan has been shown to exert positive effects on the endothelium in vitro antagonizing vascular dysfunction and inflammation. However, the function of the levosimendan metabolites within this context is still unknown. In this study, we thus investigated the impact of the metabolites OR-1896 and OR-1855 on endothelial inflammatory processes in vitro. We observed a reduction of IL-1β-dependent endothelial adhesion molecule ICAM-1 and VCAM-1 as well as interleukin (IL) -6 expression upon levosimendan treatment but not after treatment with OR-1855 or OR-1896, as assessed by western blotting, flow cytometry, and qRT-PCR. Instead, the metabolites impaired IL-1β-induced ROS formation via inactivation of the MAPK p38, ERK1/2, and JNK. Our results suggest that the levosimendan metabolites OR-1896 and OR-1855 have certain anti-inflammatory properties, partly other than levosimendan. Importantly, they additionally show that the intermediate metabolite OR-1855 does, in fact, have pharmacological effects in the endothelium. This is interesting, as the metabolites are responsible for the long-term therapeutic effects of levosimendan, and heart failure is associated with vascular dysfunction and inflammation.

## 1. Introduction

Levosimendan is a “calcium sensitizer” used in the treatment of acute decompensated heart failure (ADHF) [1,2]. Levosimendan increases the calcium sensitivity of troponin C in cardiomyocytes, thereby increasing the contractility of the heart. In addition, levosimendan increases myocardial contractility by preventing the breakdown of cAMP via the inhibition of phosphodiesterase III (PDE III) [2]. Furthermore, levosimendan induces vasodilation by opening ATP-dependent potassium channels (K(ATP)) as well as large conductance calcium-activated potassium channels (BK_Ca_) in human vascular smooth muscle cells [3,4]. Levosimendan has a short half-life of approximately one hour and a high binding affinity to serum albumin (97–98%) [5]. While the main metabolic pathway is the inactivating conjugation with glutathione before excretion [5], a small portion of the levosimendan dose (4–7%) is metabolized by intestinal bacteria to the metabolite amino-phenolpyridazinone (OR-1855) [6]. Interestingly, acetylation of OR-1855 produces the pharmacologically active metabolite OR-1896. These two metabolites can be detected approximately 12 h after infusion and have an elimination half-life of 70–80 h [5]. Although only up to 7% of the levosimendan dose is metabolized to OR-1896, this metabolite is largely responsible for the long-term effect of levosimendan on myocardial function [5]. OR-1896 was also found to induce vasodilation by activation of BK_Ca_ and K(ATP) channels [7,8] as well as inhibition of PDE III [9], similar to levosimendan.

In addition to its positive inotropic effects on cardiac function, levosimendan has also been shown to exert an anti-inflammatory effect on the endothelium. Thus, levosimendan has previously been shown to decrease cytokine-dependent expression of the pro-thrombotic factors tissue factor and plasminogen activator inhibitor type 1 (PAI-1) [10], as well as interleukin (IL) -6 and -8 and adhesion molecules such as intercellular adhesion molecule (ICAM) -1, vascular cell adhesion molecule (VCAM) -1 and E-Selectin [10,11] in endothelial cells in vitro. Mechanistically, these effects occurred by decreasing reactive oxygen species (ROS) formation and inhibiting NF-κB activity, activator protein 1 (AP-1), and hypoxia-inducible factor-1α (HIF-1α) [10,11]. Furthermore, levosimendan induces NO production in endothelial cells by activating the MAP kinases p38 and ERK [12]. Although levosimendan has been shown to affect these signalling proteins, it is unclear whether its metabolites OR-1855 and OR-1896 have similar effects on endothelial cell physiology. This is, however, of interest as the pharmacologically active metabolite OR-1896 has a much longer half-life than levosimendan and is thus responsible for the long-term effects.

Therefore, we investigated the influence of the levosimendan metabolites OR-1855 and OR-1896 on cytokine-induced cell signalling pathways, ROS formation, and adhesion molecule expression in primary endothelial cells in vitro.

## 2. Materials and Methods

### 2.1. Antibodies and Chemicals

Rabbit phospho-p38 MAPK (Thr180/Tyr182) (D3F9) XP™ (#4511), rabbit VCAM-1 (#12367), rabbit phospho-ERK1/2 (#4307), rabbit phospho-JNK (#4668), rabbit phospho-cjun (#320T), rabbit β-Actin (13E5) (#4790) antibodies were from Cell Signaling Technology (Leiden, the Netherlands). Rabbit ICAM-1 (H-108) (#sc-7891) were from Santa Cruz Biotechnology (Heidelberg, Germany). Mouse GAPDH (#MAB374) as well as anti-mouse and rabbit horseradish peroxidase-conjugated secondary antibodies (#401253 and #401353) were from Merck Millipore (Darmstadt, Germany). Mouse monoclonal APC-labelled ICAM-1 (#559771), VCAM-1 (#551147), and IgG_1_ isotype control antibody (#555751) for flow cytometry were from BD Biosciences (Heidelberg, Germany). Recombinant human IL-1β was purchased from PeproTech. OR-1855 was purchased from Hölzel Diagnostika (Köln, Germany) and OR-1896 was purchased from Hycultec (Beutelsbach, Germany). All other chemicals were from Sigma-Aldrich (Taufkirchen, Germany).

### 2.2. Human Umbilical Vein Endothelial Cell Culture

Primary human umbilical vein endothelial cells (HUVEC) were isolated from anonymous waste umbilical cords and cultivated as previously described [13]. As the cords were collected from the hospital waste, no informed consent was needed. The isolation and experiments with primary HUVEC were approved by the ethical board of the medical faculty, LMU (approval 22-0400).

### 2.3. Western Blotting

Lysates were prepared using cell lysis buffer (Cell Signaling Technology, Leiden, the Netherlands) supplemented with 10 mM PMFS and subjected to 10% SDS-PAGE following western blotting and protein band intensities quantified as described elsewhere [14]. As equal loading controls, GAPDH or actin was detected.

### 2.4. Flow Cytometry

Cell surface expression of ICAM-1 and VCAM-1 was detected as previously described [13]. In detail, HUVEC were detached from the cell culture dish using accutase upon 4 h of IL-1β (10 ng/mL) treatment, collected, and spun down for 2 min at 3000 rpm. The cell pellet was resuspended in phosphate-buffered saline supplemented with calcium (PBS+) and anti-VCAM-1, anti-ICAM-1 or IgG isotype APC labeled antibody (1:200) and incubated in the dark for 30 min. After washing with PBS+, cells were resuspended in PBS+ and adhesion molecules were detected using a BD FACS Canto (BD Biosciences, Heidelberg, Germany).

### 2.5. Quantitative Real-Time PCR

Total RNA from HUVEC was isolated using the total RNA isolation kit (VWR, Ismaning, Germany) and transcribed into cDNA using the RNA to cDNA kit (Life Technologies by ThermoFisher Scientific, Darmstadt, Germany) according to the supplier’s protocol. Quantitative PCR was performed using the PowerUp SYBR Green master mix (ThermoFisher Scientific, Darmstadt, Germany). The following primers (Metabion, Planegg, Germany) were designed and used: hIL-6 fw: 5′-ggt aca tcc tcg acg gca tct-3′ and rev: 5′-gtg cct ctt tgc tgc ttt cac-3′; human ribosomal protein S18 fw: 5′-aga aac ggc tac cac atc ca-3′ and rev: 5′-ccc tcc aat gga tcc tcg tt-3′.

### 2.6. Measurement of Reactive Oxygen Species

Intracellular ROS was detected by measuring the oxidation of H2-2′, 7′-Dichlorofluorescein (H2-DCF) to fluorescent DCF. In detail, cells were incubated with H2-DCF (20 µM) in combination with levosimendan (10 µM), OR-1855 (10 µM), OR-1896 (10 µM), inhibitors or sham (DMSO *v/v*) for 30 min followed by removal and replacement with colourless DMEM. IL-1β (10 ng/mL) was added immediately before the measurement of ROS production over 60 min at an extinction wavelength of 495 nm and an emission wavelength of 527 nm with a Tecan Spark 10 M (Crailsheim, Germany).

### 2.7. Measurement of Superoxide Radicals

NAD(P)H-oxidase dependent superoxide radical formation was measured by the superoxide dismutase (SOD) inhibitable reduction of cytochrome c as previously described [15]. In brief, endothelial cells were incubated with the eNOS inhibitor L-nitro-arginine (L-NA; 200 µM) as well as levosimendan (10 µM), OR-1896 (10 µM) or OR-1855 (10 µM) in colourless DMEM for 30 min followed by removal and replacement with colourless DMEM. IL-1β (10 ng/mL) was added in combination with 200µM L-NA and 0.5 mg/mL cytochrome c immediately before the assessment of ROS production over 60 min at 550 nm with a Tecan Spark 10M (Crailsheim, Germany). The SOD inhibitable fraction was calculated as the difference in absorbance at 550 nm between samples incubated with or without SOD (200 U/mL; Roche, Penzberg, Germany) during the measurement.

### 2.8. Statistics

Data were analyzed with Sigma Plot 10.0 (Systat Software, Inpixon, Düsseldorf, Germany). Comparison between more than two groups was performed using the One Way Analysis of Variance, ANOVA (normalized data), followed by the posthoc tests Student-Newman-Keuls Method (equal group sizes) or Dunn´s Method (unequal group sizes). For non-normalized data, the One Way Analysis of Variance on Ranks (Kruskal-Wallis), was used followed by the posthoc tests Student-Newman-Keuls Method (equal group sizes) or the Dunn´s Method (unequal group sizes). For repeated measurements of the same samples, the repeated measures ANOVA was used. Results are expressed as mean values ± SEM. Differences were considered significant at *p*-values less than 0.05 (*p* < 0.05).

## 3. Results

### 3.1. Levosimendan but Not Its Metabolites Affect the Endothelial Pro-Inflammatory Phenotype

**Figure 1:**
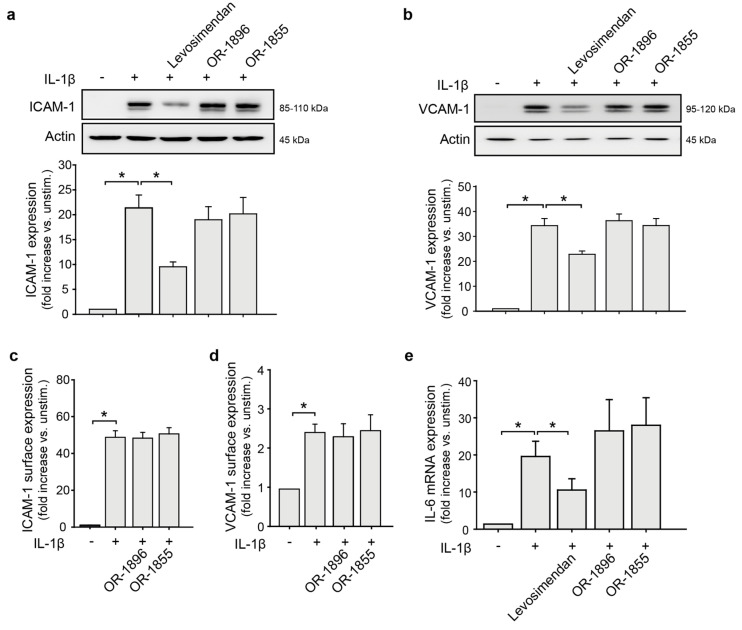
Effect of levosimendan and its metabolites on the pro-inflammatory endothelial phenotype. Endothelial cells were pre-incubated with levosimendan, OR-1855, OR-1896 (10 µM; 30 min.) or sham solution (DMSO *v*/*v*) followed by stimulation with IL-1β (10 ng/mL) for 4 h. Levosimendan reduced the expression of (**a**) ICAM-1 (n = 10; *p* > 0.05) and (**b**) VCAM-1 (n = 17; *p* < 0.05), as detected by western blot, whereas the metabolites had no effect (n = 9–10 and n = 17, respectively). The graphs show protein band densities of ICAM-1 and VCAM-1 normalized to actin (* *p* < 0.05). (**c**) IL-1β-induced cell surface expression of ICAM-1 (n = 12; * *p* < 0.05) was not affected by OR-1896 or OR-1855 treatment (n = 12). (**d**) Treatment with OR-1855 or OR-1896 (n = 14–18) did not reduce IL-1β-dependent (n = 18; * *p* < 0.05) VCAM-1 surface expression as assessed by flow cytometry. (**e**) IL-6 mRNA expression was impaired upon treatment with levosimendan (n = 12; * *p* < 0.05) but not with OR-1855 or OR-1896 treatment (both n = 12).

As it has been shown that levosimendan inhibits the expression of pro-inflammatory adhesion molecules [10,11], we first investigated if the levosimendan metabolites OR-1855 and OR-1896, in addition, influences this inflammatory response. As seen in Figure 1a,b, the IL-1β-dependent expression of ICAM-1 and VCAM-1 in endothelial cells were significantly reduced by treatment with levosimendan. In contrast, treatment with OR-1855 or OR-1896 did not influence this. To verify these results, the experiments with OR-1896 and OR-1855 were repeated and ICAM-1 and VCAM-1 surface expression was detected by flow cytometry. As seen in Figure 1c,d, none of the metabolites affected the IL-1β-induced ICAM-1 and VCAM-1 surface expression. Moreover, while levosimendan reduced the expression of IL-6 in endothelial cells, no effect was observed when applying the metabolites (Figure 1e).

### 3.2. The Metabolites OR-1896 and OR-1855 Impair Inflammatory ROS Formation

**Figure 2:**
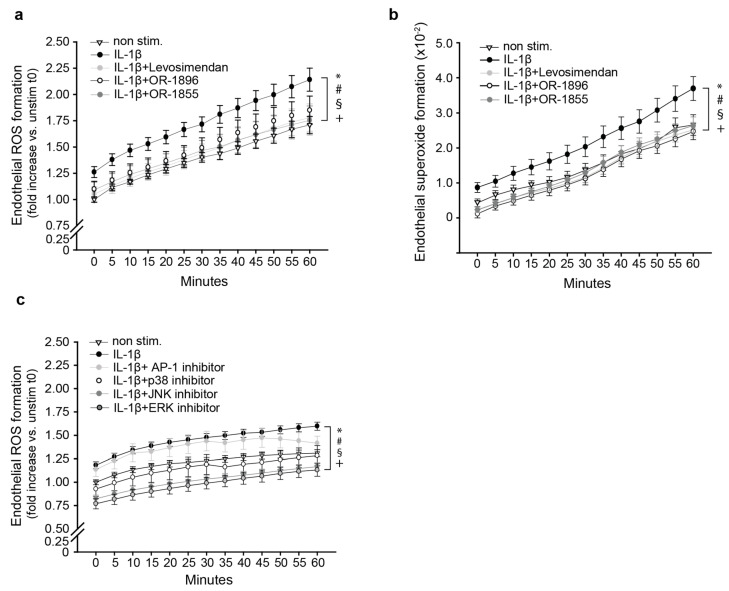
Influence of levosimendan metabolites on inflammatory ROS formation. (**a**) Pre-incubation with levosimendan, OR-1855, and OR-1896 (10 µM; 30 min), and subsequent stimulation with Il-1β (10 ng/mL) resulted in decreased ROS production in endothelial cells (all n = 7 in hexlicates; * *p* < 0.05 nonstim. vs. IL-1β at all time points; # *p* < 0.05 IL-1β vs levosimendan at 25 and 60 min; § *p* < 0.05 IL-1β vs OR-1896 at 25 and 55 min.; + *p* < 0.05 IL-1β vs. OR-1855 at 25, 55 and 60 min.), as assessed by H2-DCF oxidation. (**b**) Pre-incubation with levosimendan, OR-1855, and OR-1896 (10 µM; 30 min), and subsequent stimulation with Il-1β (10 ng/mL) impaired superoxide radical formation in endothelial cells (n = 3–6 in hexlicates; * *p* < 0.05 nonstim. vs. IL-1β 10–25 min.; # *p* < 0.05 IL-1β vs levosimendan at 0–25 min.; § *p* < 0.05 IL-1β vs OR-1896 at 0–25 min.; + *p* < 0.05 IL-1β vs OR-1855 at 0–25 min), measured by cytochrome c reduction. (**c**) Pre-treatment (30 min) with AP-1 inhibitor (S11302; 1 µM), p38 inhibitor (SB 202190; 10 µM), JNK inhibitor (SP 600125; 1 µM), and ERK inhibitor (PD 98059; 10 µM) and subsequent stimulation with Il-1β (10 ng/mL) diminished ROS production in endothelial cells (all n = 10 in hexlicates; * *p* < 0.05 nonstim. vs IL-1β at all time points; # *p* < 0.05 IL-1β vs. p38 inhibitor at all time points.; § *p* < 0.05 IL-1β vs. JNK inhibitor at all time points; + *p* < 0.05 IL-1β vs. ERK inhibitor at all time points).

The formation of reactive oxygen species (ROS) is a hallmark of endothelial activation and inflammation [16] and levosimendan has been shown to impair this response [17]. We therefore next investigated if the levosimendan metabolites OR-1896 and OR-1855 additionally affect ROS formation in endothelial cells under pro-inflammatory conditions. Whereas IL-1β stimulation increased ROS in primary endothelial cells compared to nonstimulated cells, levosimendan significantly decreased this, as previously shown (Figure 2a). Interestingly, the application of OR-1896 as well as OR-1855 also significantly impaired IL-1β-induced ROS generation (Figure 2a). Next, we measured endothelial NAD(P) H-oxidase-dependent superoxide radical formation via superoxide dismutase inhibitable cytochrome c reduction. As seen in Figure 2b, levosimendan as well as both metabolites reduced IL-1β-induced superoxide formation. To investigate the potential effect of the metabolites on underlying signalling mechanisms, we treated endothelial cells with inhibitors for the MAP-Kinases p38, ERK, and JNK before IL-1β stimulation. As seen in Figure 2c, all MAPK-inhibitors significantly prevented the IL-1β-dependent ROS formation. However, inhibition of the JNK downstream transcription factor AP-1 had no effect.

### 3.3. OR-1855 and OR-1896 Inhibit Pro-Inflammatory MAPK-Signalling in Endothelial Cells

**Figure 3:**
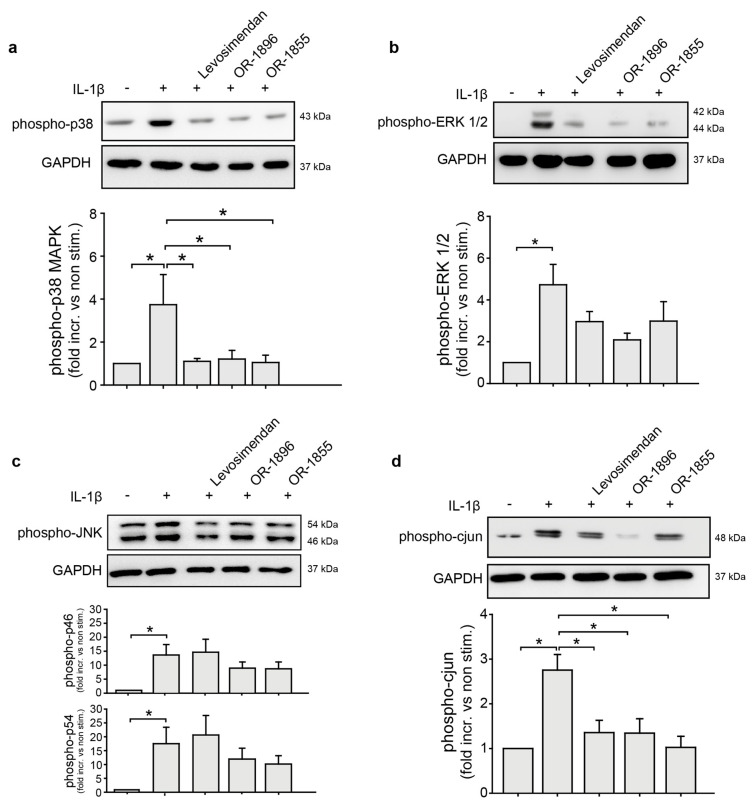
Impact of levosimendan, OR-1855, and OR-1896 on endothelial inflammatory MAPK signalling. Endothelial cells were pre-incubated with levosimendan, OR-1855, OR-1896 (10 µM; 30 min.), or sham solution (DMSO *v/v*) followed by stimulation with IL-1β (10 ng/mL) for 30 min before lysis and subsequent western blotting. (**a**) Levosimendan, OR-1855, and OR-1896 decreased IL-1β-dependent phosphorylation of p38 MAPK (n = 4). (**b**) Levosimendan, OR-1855, and OR-1896 decreased IL-1β-dependent phosphorylation of ERK1/2 MAPK (n = 4–5). (**c**) OR-1855 and OR-1896 diminished IL-1β-dependent phosphorylation of JNK (n = 15), while levosimendan had no effect (n = 15). (**d**) Levosimendan, OR-1855, and OR-1896 decreased IL-1β-dependent phosphorylation of cjun (AP-1) (n = 7). The graphs show protein band densities of phosphorylated forms of the MAP Kinases and cjun normalized to GAPDH (* *p* < 0.05).

To investigate if OR-1896 and OR-1855 affects MAPK-activation and signalling and in this way influence ROS formation, signalling activation of MAPK was assessed by detection of the respective phosphorylation. Levosimendan as well as the metabolites OR-1896 and OR-1855 impaired IL-1β-dependent p38 MAPK (Figure 3a) and ERK 1/2 activation (Figure 3b). Only OR-1896 and OR-1855 reduced JNK activation upon treatment with IL-1β, whereas levosimendan did not seem to affect this (Figure 3c). Intriguingly, levosimendan and the metabolites significantly impaired the phosphorylation of the JNK downstream target AP-1 transcription factor unit cJun (Figure 3d). As shown in Supplementary Figure S1, cJun phosphorylation was impaired by inhibition of p38 MAPK, ERK MAPK, and Akt apart from JNK, showing that AP-1 can be activated not only by JNK in endothelial cells.

## 4. Discussion

The calcium sensitizer levosimendan has shown to have extra-cardiac additional positive effects on the endothelium, such as reduction of endothelial activation and prevention of endothelial dysfunction upon inflammation [10,11,17]. The levosimendan metabolites OR-1855 and OR-1896, of which the latter is pharmacologically active, are believed to be responsible for the long-term cardiac effects of levosimendan due to their extended half-lives [5]. However, it is still unknown if OR-1855 and OR-1896 also have endothelial effects, similar to levosimendan. In this study, we thus investigated the impact of OR-1855 and OR-1896 in comparison to levosimendan on endothelial inflammation and signalling.

Chronic heart failure is linked to endothelial dysfunction, which strongly contributes to the further progression of the disease [18,19]. Endothelial dysfunction can result in endothelial activation [20], where the endothelium changes its phenotype towards a more pro-inflammatory and pro-thrombotic one with induction of adhesion molecule and cytokine expression [21] as well as enhanced production of reactive oxygen species (ROS) [22]. In patients with chronic heart failure, increased serum levels of inflammatory cytokines, such as IL-6 and TNF-α as well as the soluble adhesion molecules sICAM-1 and sVCAM-1 have repeatedly been detected [23,24,25], suggesting a vascular pro-inflammatory state. In this study, treatment of endothelial cells with levosimendan reduced the IL-1β-dependent expression of the endothelial adhesion molecules ICAM-1 and VCAM-1 as well as the pro-inflammatory cytokine IL-6. These findings are in accordance with other in vitro studies using inflammatory stimuli of endothelial cells [11,17]. Furthermore, it is consistent with clinical studies showing reduced IL-6 levels and soluble adhesion molecules in the serum of heart failure patients upon levosimendan treatment [26,27,28]. However, in our hands, neither the pharmacologically active levosimendan metabolite OR-1896 nor the intermediate metabolite OR-1855 had any influence on adhesion molecules or IL-6 expression in endothelial cells. In contrast, treatment with the two metabolites reduced IL-1β-mediated endothelial ROS formation, similar to levosimendan. Indeed, while the impact of levosimendan on ROS generation in endothelial cells have previously been detected by others [17], the observed effect of OR-1855 and OR-1896 in this study was not previously known. The exact underlying mechanism of ROS inhibition of levosimendan and its two metabolites, however, still remains to be investigated. We observed that IL-1β-induced ROS production in endothelial cells could be prevented by inhibiting the MAPK ERK1/2, p38, and JNK. Moreover, OR-1855 and OR-1896 reduced the phosphorylation and thus activation of ERK1/2, p38, and JNK. This suggests that the levosimendan metabolites may inhibit inflammatory ROS formation by preventing MAPK-signalling activation. MAPK are known to become activated by oxidative stress [29] but have also been described to influence ROS formation in certain conditions by for instance enhancing mitochondrial superoxide generation [30,31] or via activation of the NAD(P)H-oxidase [32]. Indeed, levosimendan as well as both metabolites impaired endothelial NAD(P)H-oxidase-dependent superoxide radical formation in our hands. Intriguingly, whereas our results showed reduced p38 MAPK and ERK1/2 activation upon levosimendan treatment, as in line with previous results by others [12], we did not observe any effect of levosimendan on JNK activation. Moreover, despite this, levosimendan impaired the phosphorylation of the AP-1 transcription factor subunit cjun, which is a downstream target of JNK. This somewhat controversial finding may be explained by the fact that we observed activation of cjun by other MAPK apart from JNK in endothelial cells, suggesting that levosimendan influences cjun activation rather via p38 MAPK or ERK 1/2. This activation of cjun has been described before [33,34,35]. However, AP-1 inhibition did not influence IL-1β-induced ROS formation. This suggests that the reduction in cjun phosphorylation and thus AP-1 transcriptional activity by levosimendan as well as the metabolites may influence other processes in endothelial cells, such as cell survival.

Intriguingly, although MAPK has also been demonstrated to be important for the expression of adhesion molecules [36,37], we did not detect an effect of the metabolites. However, in a previous study, we demonstrated that IL-1β-dependent ICAM-1 and VCAM-1 expression in HUVEC was mediated by p38 MAPK but not by ERK1/2 or JNK, showing differential regulation of these adhesion molecules by the different MAPK in endothelial cells [38]. In addition, inhibition of AP-1 did not have any effect on adhesion molecule expression under the same conditions [38]. The finding that OR-1896 and OR-1855 influence p38 MAPK activity but not ICAM-1 or VCAM-1 expression in contrast to levosimendan, which affected p38 MAPK as well as adhesion molecule expression, may be due to differential activation of downstream targets of p38 MAPK. Differential activation of cellular feedback mechanisms by levosimendan and the metabolites may also be possible and explain the different responses. The exact mechanisms hereof and if OR-1896 and OR-1855 inhibit the upregulation of other pro-inflammatory or pro-coagulatory proteins need to be investigated in further studies. In addition, if OR-1896 and OR-1855 may have effects on chronic endothelial inflammation remains to be investigated.

Of note, we not only observed a so far undescribed effect of the pharmacologically active levosimendan metabolite OR-1896, but we additionally detected an effect on IL-1β mediated processes by the intermediate metabolite OR-1855, which has so far been described as pharmacologically inactive. As OR-1855 and OR-1896 have much longer half-lives compared to levosimendan (70–80 h versus 1–2 h), the discovery of effects on the endothelium by these metabolites is interesting and may be clinically relevant. As patients with cardiac insufficiency present with elevated cytokine levels [24,25] and vascular dysfunction including a pro-inflammatory state [39] with enhanced ROS production [40], levosimendan treatment may in addition to its positive effects on cardiac contractility exert protective effects on the endothelium thereby reducing the risk of cardiovascular events.

Furthermore, levosimendan is also used in the perioperative setting of cardiac surgery in combination with cardiopulmonary bypass [41,42], where up to a third of patients may develop a systemic inflammatory response syndrome (SIRS) in response to the surgery [43], which is a serious condition that can lead to organ failure and death [44]. As the endothelium is essential in the progression of SIRS-induced organ failure, an additional protective effect of the long-lasting metabolites may thus be advantageous within this context. Especially, as these patients also experience ischemia-reperfusion injury after removing the cardiopulmonary bypass with increased formation of ROS [45,46], levosimendan treatment may be additionally advantageous in this situation. However, the concentrations of levosimendan used in this and most in vitro studies (10 µM) are relatively high and may not reflect the actual serum levels in patients treated with levosimendan. Especially regarding the metabolites when considering that only up to 7% of the levosimendan dose is metabolised to OR-1855 and OR-1896. To elucidate if the endothelial protective effects observed here are clinically relevant, studies measuring the serum levels of levosimendan and its metabolites in patients with (perioperative) application of levosimendan with subsequent assays using the detected serum concentrations in cell culture need to be performed. Indeed, these are the aims of further studies by us.

In summary, we show that the levosimendan metabolites OR-1855 and OR-1896, in contrast to levosimendan, do not affect endothelial inflammatory adhesion molecule expression but cytokine-induced MAPK activation leading to cellular ROS formation. Importantly, we not only describe the novel effects of OR-1855 and OR-1896 in endothelial cells but additionally show that the pharmacologically inactive intermediate metabolite OR-1855 does indeed have an effect on endothelial processes. These results may be interesting in the context of levosimendan treatment of patients with cardiac failure in light of the long-term effects of levosimendan, which originates from the metabolites. The clinical relevance, however, remains to be investigated.

## Data Availability

The data sets generated during the current study are available from the corresponding author on reasonable request.

## Supplementary Materials

The following supporting information can be downloaded at: https://www.mdpi.com/article/10.3390/biomedicines11030918/s1, Figure S1: Investigation of kinases upstream of cjun.
