# Supplementary material for: The Effects of the Levosimendan Metabolites OR-1855 and OR-1896 on Endothelial Pro-Inflammatory Responses
Source: Biomedicines. 2023 Mar 16;11(3):918. doi: 10.3390/biomedicines11030918 (PMC10045601; doi:10.3390/biomedicines11030918)
Supplement: Supplementary file 1 [file biomedicines-11-00918-s001.zip › biomedicines-2132659-supplementary.pdf]

Supplementary Materials

The Effects of the Levosimendan Metabolites OR-1855 and OR-1896 on Endothelial Pro-Inflammatory Responses

Hannah Kipka, Rebecca Schaflinger, Roland Tomasi, Kristin Pogoda and Hanna Mannell

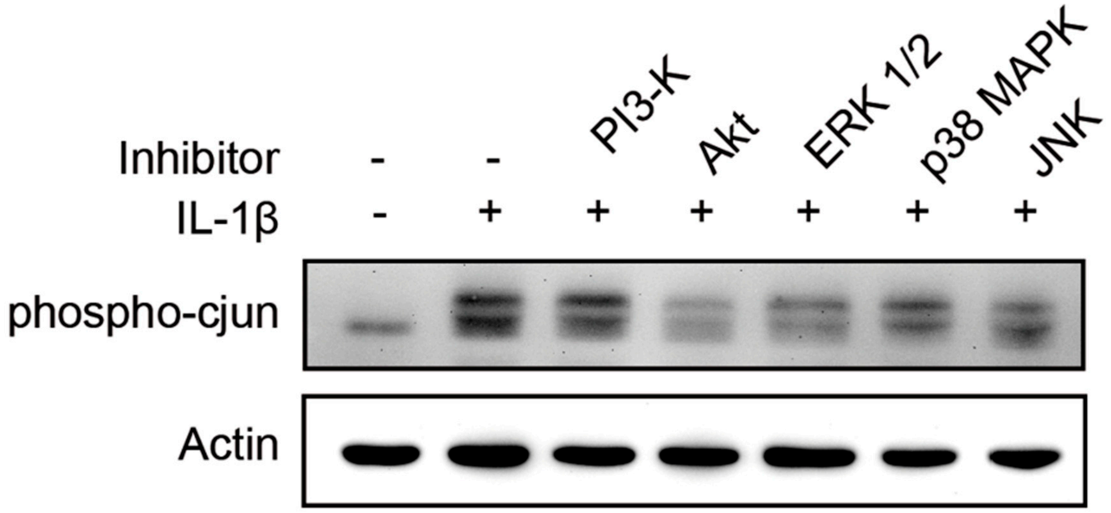

Figure S1. Investigation of kinases upstream of cjun. Endothelial cells were pre-incubated (30 min.) with PI3-K inhibitor (Ly294002; 10 µM; n=2), Akt inhibitor (10 µM; n=5), ERK 1/2 inhibitor (PD 98059; 10 µM; n=5), p38 MAPK inhibitor (SB 202190; 10 µM; n=2), JNK inhibitor (SP 600125; 1 µM; n=5) or sham solution (DMSO v/v; n=5) followed by stimulation with IL-1β (10 ng/ml) for 30 min before lysis and subsequent western blotting.
